# Instant interaction driven adaptive gaze control interface

**DOI:** 10.1038/s41598-024-62365-9

**Published:** 2024-05-22

**Authors:** Kun Qian, Tomoki Arichi, A. David Edwards, Joseph V. Hajnal

**Affiliations:** King’s College London, Centre for the Developing Brain, School of Biomedical Engineering and Imaging Sciences, London, SE1 7EH UK

**Keywords:** Computer science, Biomedical engineering

## Abstract

Gaze estimation is long been recognised as having potential as the basis for human-computer interaction (HCI) systems, but usability and robustness of performance remain challenging . This work focuses on systems in which there is a live video stream showing enough of the subjects face to track eye movements and some means to infer gaze location from detected eye features. Currently, systems generally require some form of calibration or set-up procedure at the start of each user session. Here we explore some simple strategies for enabling gaze based HCI to operate immediately and robustly without any explicit set-up tasks. We explore different choices of coordinate origin for combining extracted features from multiple subjects and the replacement of subject specific calibration by system initiation based on prior models. Results show that referencing all extracted features to local coordinate origins determined by subject start position enables robust immediate operation. Combining this approach with an adaptive gaze estimation model using an interactive user interface enables continuous operation with the 75th percentile gaze errors of 0.7^∘^, and maximum gaze errors of 1.7^∘^ during prospective testing. There constitute state-of-the-art results and have the potential to enable a new generation of reliable gaze based HCI systems.

## Introduction

In human-computer interaction (HCI), people-oriented strategies form a key design principle that focuses on interaction interface design to minimise the workload for users. Instant control, by which the user can immediately start productive system use is important for users, especially young children, because it makes the interaction with technology easy, fast and intuitive. This kind of control is a familiar feature of a computer mouse and other physical interaction control systems, but is not generally available for indirect, non-contact methods, which frequently require a calibration step prior to each user session. One such HCI is gaze control, a technique that involves use of the inferred point of visual focus by the user as they look at a screen or scene to select and manipulate computer functions. This requires real time monitoring and analyzing of eye features (such as pupil and corneal reflection data) to compute the gaze location. However, the current state of gaze control technology falls short in terms of being instantly and effortlessly usable as existing methods are not capable of providing immediate reliable gaze estimation^1,2^.

Gaze estimation requires a model to compute the fixation point on the screen from the eye feature data, which usually needs to be personalised to accommodate individual-specific characteristics and variations. However, building personalised models can be challenging if the geometry is not tightly controlled, due to a requirement for training data that is sufficient to accommodate all possible variations in eye features and head poses. In many cases this is not practically feasible^3^. To achieve operational performance, it is therefore common to deploy a calibration procedure^2^ which typically requires the user to stare at multiple calibration points distributed across the field of view (normally 9 points), watch a video or perform extra user tasks (e.g. mouse clicks or target pursuit) to obtain data to build the personalised model. Despite personalised calibration, the resulting models still tend to be vulnerable to failure caused by even small changes in head position^2^. In addition, the calibration procedure itself is usually very tedious and requires user cooperation making it challenging to perform or unsuitable for particular populations, especially younger users or those with cognitive or physical impairments.

Developing a gaze model procedure that can adapt to individual specifics while maintaining a consistent level of accuracy and reliability across all users is challenging. Among gaze estimation methods, the 2D feature mapping (regression) based methods are widely used for real-time applications due to their simplicity and reasonable accuracy. They establish a mapping between 3D gaze direction or 2D gaze coordinates and an eye feature or features, including gaze direction related features (e.g. pupil/iris, corneal reflection positions)^2^ and head motion/pose related features (e.g. eye corner, rigid facial features positions)^4,5^ which are represented in a global coordinate system. However, representing features in a global coordinate system makes the mapping dependent on absolute values, which can vary significantly across individuals. For instance, the same gaze direction can be associated with different feature values from one person to another due to differences in disposition of the eyes in the face and variations in head pose^6^. Thus, without additional supervision, it is difficult to learn this variability. Very few approaches have been described that offer approaches which are robust against individual differences yet are suitable for low cost systems (i.e., without a requirement for a complex lighting setup) while allowing head motion^5^.

In this paper, we propose an eye feature construction method based on local relative coordinate systems rather than global absolute space representations and explore the potential of initialising models with a pre-acquired database to allow the subject to achieve instant control for HCI . Here we define instant control as only requiring the subject to look at a single target presented on an opening screen - analogous with checking the initial pointer location when first putting a hand on a computer mouse. We present and test a combination of immediate control and adaptive modelling, in which the act of using gaze based interaction provides further data to update the gaze estimation model. The aim is to achieve instantaneous and continuous gaze control that is comfortable for the user from the very start of their HCI session. The motivation for this work was our separate development of virtual reality (VR) entertainment techniques using gaze control for subjects undergoing brain examination by MRI^7^. For such systems to be effective, non-expert subjects need to be able to get immersed and be in control with minimal pre-amble or training and, to avoid strain, the gaze tracking needs to remain accurate and robust for prolonged periods of continuous use. This paper describes our exploration of ways to remove the need for explicit calibration tasks, ideally achieving an “instant on” capability that enables seamless VR control as soon as the subject starts to look at the screen. As young children have a high need for immediate and continuous use, particularly in the context of an MRI examination, our testing included this group, with the youngest participant being 2 years of age. Key system components studied are choice of coordinate origin when instantiating gaze tracking models, use of multi-subject prior datasets to build initialisation models and adaptive model updates that can achieve resilience to changes in pose.

## Related works

To achieve instantaneous and continuous gaze interaction, existing research has centred around the development of gaze models that possess the capability to adapt to individual specifics while maintaining a consistent level of accuracy and reliability across all users. Their focus has been on gaze models and/or new calibration workflows.

### Gaze model

Gaze estimation methods can be generally categorized into geometric based, 2D feature mapping based and appearance based^2^. Geometric-based methods take a full 3D approach utilizing eye-in-head rotations and head-in-world coordinates to calculate a gaze vector and its intersection with a planar display. This is the most accurate method but normally requires a complex lighting and camera setup to infer eye geometry and thus is not suitable for use in dynamic and complex environments. The 2D feature mapping based methods estimate gaze by building a mapping function between 2D eye features extracted from live video images and gaze point on a screen. Common features include: pupil/iris-corner, pupil/iris-corneal reflection, cross-ratio (CR), and homography normalization (HN)^2^. The construction of the function mapping from feature to gaze generally requires explicit personal calibration, with the learned mapping often being vulnerable to even small head motion. Appearance-based methods directly map eye appearance images to desired gaze coordinates or directions, making them more suitable for complex and dynamic scenarios. However, appearance-based methods are sensitive to environmental factors and feature variations, demanding extensive data and long offline training times. Recent studies in appearance-based gaze estimation have focused on the development of generalizable gaze estimation methods which are less affected by gaze-irrelevant factors. For example, gaze purification^8,9^ aims to minimize gaze-irrelevant factors (environment or facial appearance) and extract the intrinsic gaze pattern that remains consistent across all domains. However, it still focuses on absolute global frame appearance features, so can be vulnerable to individual variations such as for people who naturally looks slightly upward which can result in their gaze being misinterpreted as consistently higher than it actually is.

The 2D feature mapping based methods are widely used for real-time applications due to their simplicity and reasonable accuracy. These approaches have two key aspects: constructing eye features and choosing an appropriate mapping function. Reflection-based features are among the most commonly used eye features but rely on infrared light sources and user cooperation to keep the reflection on the corneal surface throughout use. As a result, the pupil centre-eye corner (PCEC) feature, which is considered to be partially equivalent to the pupil centre-corneal reflection (PCCR) vector, has been proposed in^10^. However, the PCEC feature only provides an estimation of eyeball pose and fails to account for changes in head pose. This can be mitigated by integrating information about head motion, such as rigid facial feature points (e.g. inner and outer eye corner, nose tip) with the PCEC into an augmented feature vector^4,5^. Other methods use head motion information to correct mapping results^11^ or normalize the iris centre-eye corner vector to account for changes in pose^12^. A more sophisticated normalization workflow was later introduced by Zhang et al. (2018)^6^, which relies on understanding the relationship between face pose and camera pose, and thus requires complete facial information.

A wide range of mapping functions have also been proposed, including convolutional neural networks^5^, Support Vector Machines (SVM)^13^, and polynomial equations^14^). A common feature of work to date is to represent eye features in a global space which encodes rather than removes individual variation. Currently, there is limited research dedicated to constructing eye features that capture relative movements and reveal universal patterns of gaze changes in response to head movements.

### Implicit calibration

To make the calibration less tedious and improve user experience, researchers have proposed implicit calibration. Early studies on implicit calibration relied on manual input such as a mouse click or key press^15–18^ to infer gaze location. However, those methods require external hardware and users had to perform click tasks to collect sufficient samples for robust interaction (e.g. 1000 clicks, 1500 clicks, 554 clicks achieve a gaze error around 4^∘^^16^, 2.5^∘^^17^ and 5^∘^^18^ respectively). A more general solution based on visual saliency maps was proposed in^19–21^ to estimate gaze location probability. Eye image and saliency map pairs are extracted while subjects watch a video clip or view images (training on 7500 video frames achieved a 3^∘^ error^19^). Pursuit calibration^22–24^ is based on smooth pursuit eye movement when following a moving object. However, these approaches still require user cooperation and additional tasks, with a 10-s target pursuit achieving a 1^∘^ error^23^. For participants where this may be more challenging like those with attention difficulties^24^, a 30–60 s pursuit procedure achieves about 2^∘^ error. The coordination of hand-eye movements during interaction has been extensively studied in neuroscience^25^. Sidenmark et al.^26^ examined implicit calibration during hand interactions in VR, such as release, reach, and manipulation. Such methods can achieve 1^∘^ error with 1 min of operation (300+ gaze samples collected). Furthermore, although one-point calibration is user-friendly, it requires a fully calibrated device^27^. In addition, knowledge of camera, light source, and monitor positions^1^ is impractical in many less well controlled environments. Cross-ratio based methods^28–32^ reduce dependency on calibration but constrain user behavior and lack practicality in various scenarios because the dependency on complex light setup.

Thus although there is a substantial literature on gaze estimation and its use in HCI, practical deficiencies that inhibit wide uptake of this technology remain. From the user perspective, having to endure a calibration process, perhaps with manual adjustment of software settings with each new session makes a poor alternative to robust physical interaction devices (e.g. a computer mouse) that require just an initial slight movement to establish hand-eye coordination and get working. The need for users to remain in a fixed pose for prolonged operation is also a major practical limitation, particularly for specific populations, such as children, where this is likely to be challenging.

## Gaze estimation

The current gaze location, defined as a 2D coordinate on the target screen, ${\textbf {g}} = (gx, gy)$, is estimated from an eye feature vector, **e**, via model *f*, such that ${\textbf {g}} = f({\textbf {e}})$. We adopted the widely employed quadratic polynomial-based approach^14^ for gaze estimation, also known as 2D regression-based gaze estimation. However, the conventional polynomial-based methods typically use a definition of ${\textbf {e}}$ based on the measured coordinates of the pupil alone or pupil-cornea reflection (or eye corner) vector (${\textbf {e}} = (x,y)$, where (*x*, *y*) are coordinates or vectors in an image space such as a live video feed showing the eyes) and thus cannot account for the impact of head motion. To address this limitation, head motion can be integrated into ${\textbf {e}}$ as a facial feature vector (*m*, *n*) (e.g. rigid facial feature point) used to detect changes in head pose^4,5,7^. We use eye corner coordinates for (*m*, *n*) in the method described here, which is a widely used alternative to corneal reflection^10^. We construct the eye feature as ${\textbf {e}} = (x,y,m,n)$. The gaze estimation model can then be formulated in matrix notation as ${\textbf {g}}=f({\textbf {e}})={\textbf {vc}}$, and where ${\textbf {v}} =(1,x,y,xy,x^2,y^2,m,n)$ assembles terms up to quadratic order in the elements of ${\textbf {e}}$ and ${\textbf {c}}= ({\textbf {c}}_x,{\textbf {c}}_y)$, where ${\textbf {c}}_x=(a_0,a_1,a_2,a_3,a_4,a_5,a_6,a_7)^T$, ${\textbf {c}}_y=(b_0,b_1,b_2,b_3,b_4,b_5,b_6,b_7)^T$ contain the unknown model coefficients which can be solved separately. To solve for the coefficients ${\textbf {c}}$, calibration data or a training set is needed which is composed of several measured eye feature-target pairs $({\textbf {e}}_i, {\textbf {t}}_i)$, where ${\textbf {t}}_i=(tx_i,ty_i)$ is the target location on the screen that is gazed at when ${\textbf {e}}_i$ is recorded. We call the paired data obtained during subject specific calibration a *gaze fixation sample*. A common strategy is to obtain subject specific calibration data at the start of each user session of the gaze estimation system by requiring users to fixate at a sequence of M targets $({\textbf {t}}_1, {\textbf {t}}_2, \ldots {\textbf {t}}_M )$ on the screen. For each eye feature vector ${\textbf {e}}_i$ where $i = 1,2,3\ldots M$, a corresponding ${\textbf {v}}_i$ can be calculated. The gaze model can thus be formulated as:

1 $$\begin{aligned} {\textbf {T}} = ({\textbf {t}}_1, {\textbf {t}}_2, ..., {\textbf {t}}_M)^T = \begin{pmatrix} {\textbf {v}}_1 \\ ...\\ {\textbf {v}}_m \end{pmatrix}{} {\textbf {c}} = {\textbf {V}}{} {\textbf {c}} \end{aligned}$$

A least squares gaze model can be solved as:

2 $$\begin{aligned} {\textbf {c}} = ({\textbf {V}}^T{\textbf {V}})^{-1}{} {\textbf {V}}^T{\textbf {T}} \end{aligned}$$

Here we replace the calibration procedure using a prior model which is made up of eye feature-target pairs previously collected beforehand from different subjects. Although the model is therefore not personalized, it can fulfill the aim for the user not to have to complete any calibration procedure. An important consideration is that the subject head pose as recorded on the live video will inevitably vary between trials and individual subjects also have variable spatial dispositions of the features that comprise ${\textbf {e}}$. Multi-subject paired (${\textbf {e}}_i,{\textbf {t}}_i$) data can be used to solve for the same type of model, ${\textbf {c}}$, but to increase flexibility we introduce weights, ${\textbf {W}}$, for the measurements from each subject that contributes data to the gaze fixation sample, leading to Eq. (3).

3 $$\begin{aligned} {\textbf {c}}=({\textbf {V}}^T {\textbf {W}}^T {\textbf {W}}{} {\textbf {V}})^{-1} {\textbf {V}}^T {\textbf {W}}^T {\textbf {W}}T \end{aligned}$$

**Table 1:** Definitions of coordinate spaces and eye features, and description of related features from a geometric perspective.

Designation of coordinate spaces	Feature definition	Feature descriptions	Coordinate origin(s)
Global absolute	${\textbf {e}}_i=(x_i, y_i, m_i, n_i)$	Absolute pupil position and absolute head position	Global original
Global relative	${\textbf {e}}_i=(x_i - m_i, y_i - n_i, m_i, n_i)$	Pupil pose relative to eye corner combined with absolute head position	Global original
Corner absolute	${\textbf {e}}_i=(x_i- m_0, y_i- n_0, m_i-m_0, n_i-n_0).$	Both pupil and eye corner relative to a fixed origin located at an initial eye corner position	Eye corner
Corner relative	${\textbf {e}}_i=(x_i- m_i, y_i- n_i, m_i-m_0, n_i-n_0).$	Pupil pose relative to the eye corner corrdinate, and local eye corner coordinate relative to an intial eye corner location	Eye corner
Local absolute	${\textbf {e}}_i=(x_i- x_0, y_i- y_0, m_i-m_0, n_i-n_0).$	Both pupil and eye corner specified relative to their own initial recorded positions	Pupil and eye corner
Local relative	${\textbf {e}}_i=(dx_i, dy_i, m_i-m_0, n_i-n_0)$ where $dx_i=(x_i- x_0)-(m_i- m_0), dy_i=(y_i- y_0 )-(n_i- n_0).$	Pupil pose relative to local eye corner positon with both indexed to their own initial recorded positions	Pupil and eye corner

$(x_i,y_i)$ and $(m_i,n_i)$ are the ith recorded pupil and eye corner positions. For global absolute and relative space, all measurements for all subjects are based on the same fixed global origins. For eye corner space, the first measured eye corner coordinate $(m_0,n_0)$ for each subject is used as a fixed origin for that subject. For local space, both pupil and eye corner measurements for each subject are referenced to the first recorded position of each, $(x_0,y_0 )$,$(m_0,n_0)$, for that subject.

## Coordinate space representation

**Figure 1:**
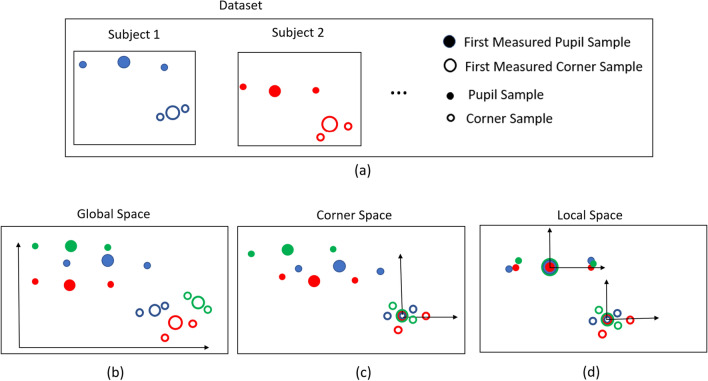
Comparison of global, corner space and local space based eye feature representation. (**a**) Illustrates the eye features (in global space) for each subject included in the dataset (training data) and presents the graphical definition of first measured pupil/corner sample, and other pupil/corner samples. (**b–d**) Depict the eye feature distributions in global space, corner space, and local space respectively. The green-colored eye features in (**b–d**) Represent test eye feature data (test data) that are not part of the initial dataset (training data).

For each eye, the pupil coordinates (*x*, *y*) and the eye corner coordinates (*m*, *n*) are extracted from a live video stream, with both features measured for each video frame in pixel units with origin at the centre of the video image. Likewise, the target coordinates *tx* and *ty* are measured in screen pixel units with origin at the centre of the display screen. We explore three choices of coordinate origin for the elements of ${\textbf {e}}$ which will be referred to as global, corner and local (see Table 1). In each case, the eye feature is modelled either using absolute coordinates (pupil only) or relative to the eye corner (pupil-corner vector). We use the terms “absolute” to mean that the origin is at a fixed location for the duration of an examination, and “global” to indicate that the choice of origin is independent of image content (e.g. the centre of the pixel grid). Figure 1 compares the eye feature distributions resulting from different choices of origin. The use of a global origin preserves all subject feature locations so is more sensitive to variations introduced by facial features and resting head pose (Fig. 1b). Adopting session specific origins (local eye corner origins) reduces variability (Fig. 1c) and using separate local origins (pupil and corner origins) for each element of ${\textbf {e}}$ in each session further reduces variability between subjects (Fig. 1d).

## Adaptive model update

We distinguish between static gaze models that remain fixed and adaptive models^7^ that can adjust during a user session. Both types of model can be initialised using conventional calibration (gaze fixation sample) or prior model data provided there are screen location samples that span the desired work-space. As the subject is usually instructed to remain still during calibration it is likely that only a single or very limited range of head poses will have been sampled. Thus a clear potential benefit of the prior model approach is that it includes more diversity in head pose. The performance of static models is likely to be affected by changes in head position during normal operation. Adaptive gaze modelling^7^ has the potential to achieve continued accuracy when there is a change in head pose, and can increase robustness to sudden head motion by achieving a personal model that is informed dynamically during the current session. We therefore explore an adaptive model update method. This can be achieved using Eq. (3) by simply by adding new gaze fixation samples collected as part of the current user session. The approach can be applied using a conventional calibration phase or starting with the prior model data from multiple subjects. A key requirement is a suitable user interface that allows recording of $({\textbf {e}},{\textbf {t}})$ pairs as an integrated part of normal usage.

**Figure 2:**
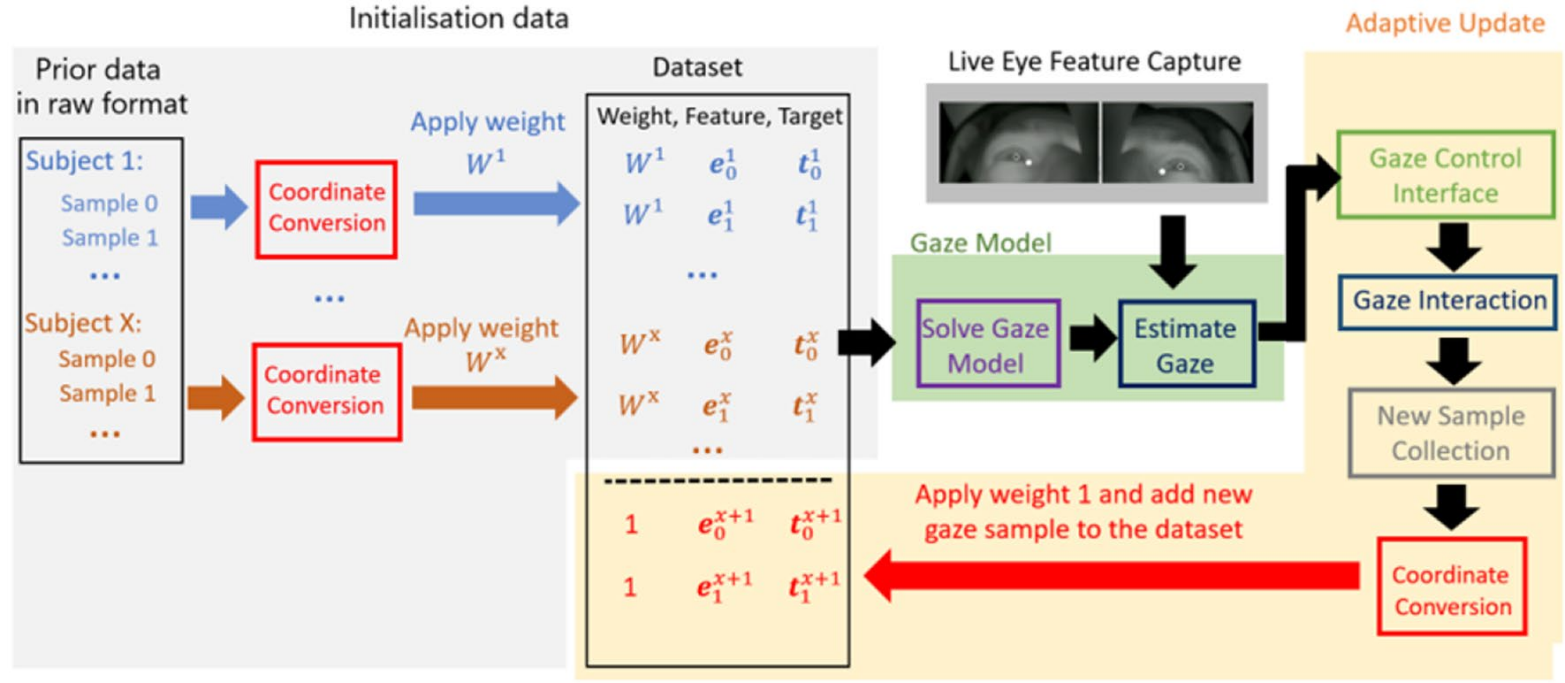
The full gaze estimation pipeline including optional adaptive model update. The coordinate conversion applied can be any one from Table 1. Gray shaded region: preparation of the initial data either from prior measurement samples or from a calibration phase; green shaded section: deployment of the current gaze estimation model; yellow shaded section: optional adaptive update.

In detail, as can be seen in Fig. 2, the approach involves the following steps: (1) Coordinate conversion to reference all data to a given choice of origin as described above. We denote the *j*th subject’s *i*th measurement eye feature as: ${\textbf {e}}_i^j$, with ${\textbf {e}}_0^j$ being the first measured feature in their examination. (2) Initial model construction using calibration samples as a prelude to the session or gaze fixation samples collected from other subjects beforehand. (3) Gaze estimation and interaction during any given session. The estimated gaze will be sent to the gaze control interface to interact with specific content. For the corner and local space based coordinates, the origin of coordinates is determined from the first measured gaze fixation sample $e_0^j$. (4) Adaptive model update. When this feature is enabled, the gaze fixation samples collected from the current subject are added to the initial dataset and the gaze estimation model is recalculated using the weighted least square method in Eq. (3). To ensure that measurements from the current user rapidly dominate the gaze model, incoming data is given a much larger weight than the data from prior subjects.

## Initialising the gaze estimation model

When using the eye corner and local coordinate systems it is necessary to initialise the system with a first measurement, $${\textbf {e}}_0$$, for the current subject. The software automatically streams the elements of $${\textbf {e}}$$ whenever the camera feed contains detectable eyes. To ensure robust starting conditions each session starts with a single target on the screen. To be consistent with the gaze fixation samples included in the initial dataset, we adopt a convention that the origin of coordinates should correspond to the subject looking at the screen centre. For most testing, we simply used a target at the screen centre, $${\textbf {t}}_c=(tx_c,ty_c)$$. However, the method supports use of an arbitrary initial target $${\textbf {t}}_a=(tx_a,ty_a)$$. This can be achieved by solving (or reconstructing) the eye feature corresponding to a screen centre target using the inverse of prior model given the actual target location. An arbitrary initial target $${\textbf {t}}_a$$ and its corresponding pupil position (*x*, *y*) and eye corner position (*m*, *n*), must satisfy: $${\textbf {f}}(x- x_0,y- y_0,m-m_0,n- n_0 )= {\textbf {t}}_a$$, where $$(x_0,y_0)$$ and $$(m_0,n_0)$$ are the pupil and eye corner coordinates that relate to the screen centre position $${\textbf {t}}_c$$ at the current head pose (so $$m= m_0,n= n_0)$$. As the gaze estimation model f is quadratic, the two unknowns, $$(x_0 y_0)$$ can be determined using Eq. (4), which we solve numerically using an iterative solver with a termination tolerance of 1e−8. Recognizing the potential for multiple solutions due to the quadratic nature of the gaze estimation model, we need to choose a valid solution. The resolution of the eye tracker’s input image is 640 $$\times $$ 480 pixels, with the eye region occupying approximately 160 $$\times $$ 100 pixels. So we select the only solution which lies within a 200 $$\times $$ 200 pixels rectangular region centered at (x, y). Solutions outside this region are deemed unphysical or are complex in nature, and hence are disregarded.

4 $$\begin{aligned} (x_0,y_0)=(x,y)- f^{-1} ({\textbf {t}}_a) \end{aligned}$$

The reconstructed gaze fixation sample $({\textbf {e}}_0,{\textbf {t}}_c) = (x_0,y_0,m_0,n_0,tx_c,ty_c)$ and the gaze sample measured for the arbitrary initial selection target $({\textbf {e}}_1,{\textbf {t}}_a)= (x,y,m,n,tx_a,ty_a)$ are then both added to the dataset and any required local coordinate system is constructed using ${\textbf {e}}_0$ for the current subject.

All work was approved by the King’s College London research ethics office (reference: LRS/DP-21/22-33239) or NHS health research authority (reference: 18/LO/1766) and carried out in accordance with institutional guidelines including informed consent from the participants and/or guardians.

## Experiment design

### Experiment platform

The primary data used throughout this study consists of gaze fixation samples $({\textbf {e}},{\textbf {t}})=(x,y,m,n,tx,ty)$, which are used to explore and analyse the effectiveness of our methods in achieving accurate, robust and stable gaze estimation without the need for an explicit calibration target sequence. We collected gaze fixation samples based on our adaptive gaze tracking based VR platform with content designed for patient distraction and/or entertainment during MRI scanning^7^. The pupil and corner features are extracted using a customized eye tracking algorithm, built upon the widely recognized deep learning framework, YOLOv5^33^. This is required in the present study because full facial information is not available in the captured videos as a result of physical occlusion by the presence of the MRI coil. However, this was an added requirement in preparing our system rather than a limitation. The adaptive gaze system we describe is dependent on robust extraction of the eye features we exploited and is not restricted to any specific method of feature extraction. It is compatible with any face tracking techniques that can provide pupil and eye corner or rigid facial feature information. Importantly, the choice of segmentation method is not a critical feature of the presented material.

The restricted field of view of the camera added challenge, but consistency of image content and stability of lighting conditions resulting from exclusion of ambient light by the head coil and main scanner itself reduced diversity in the scene to be segmented. Notably, our eye images are not subject to the complex and variable conditions typically encountered in outdoor eye tracking scenarios^34^. However, in some subjects, particularly children, it was possible for the contralateral eye to be visible in the captured videos. The analysis region was limited to ensure there was only one set of eye features per camera image (In consequence, at run time it is occasionally necessary to make a single manual adjustment to the medial limit of this region). We adopted a progressive approach. We annotated the pupils and eye corners in an independent dataset, progressively testing on held out data, and found that after ~2000 annotated examples results were stable. The final training set consisted of 2,936 images collected from 34 subjects. Additional diversity was introduced into the training set using flip, brightness, exposure and blur operations (using Roboflow^35^) to achieve a total of 6607 annotated. There were no feature extraction failures throughout the test phase.

**Figure 3:**
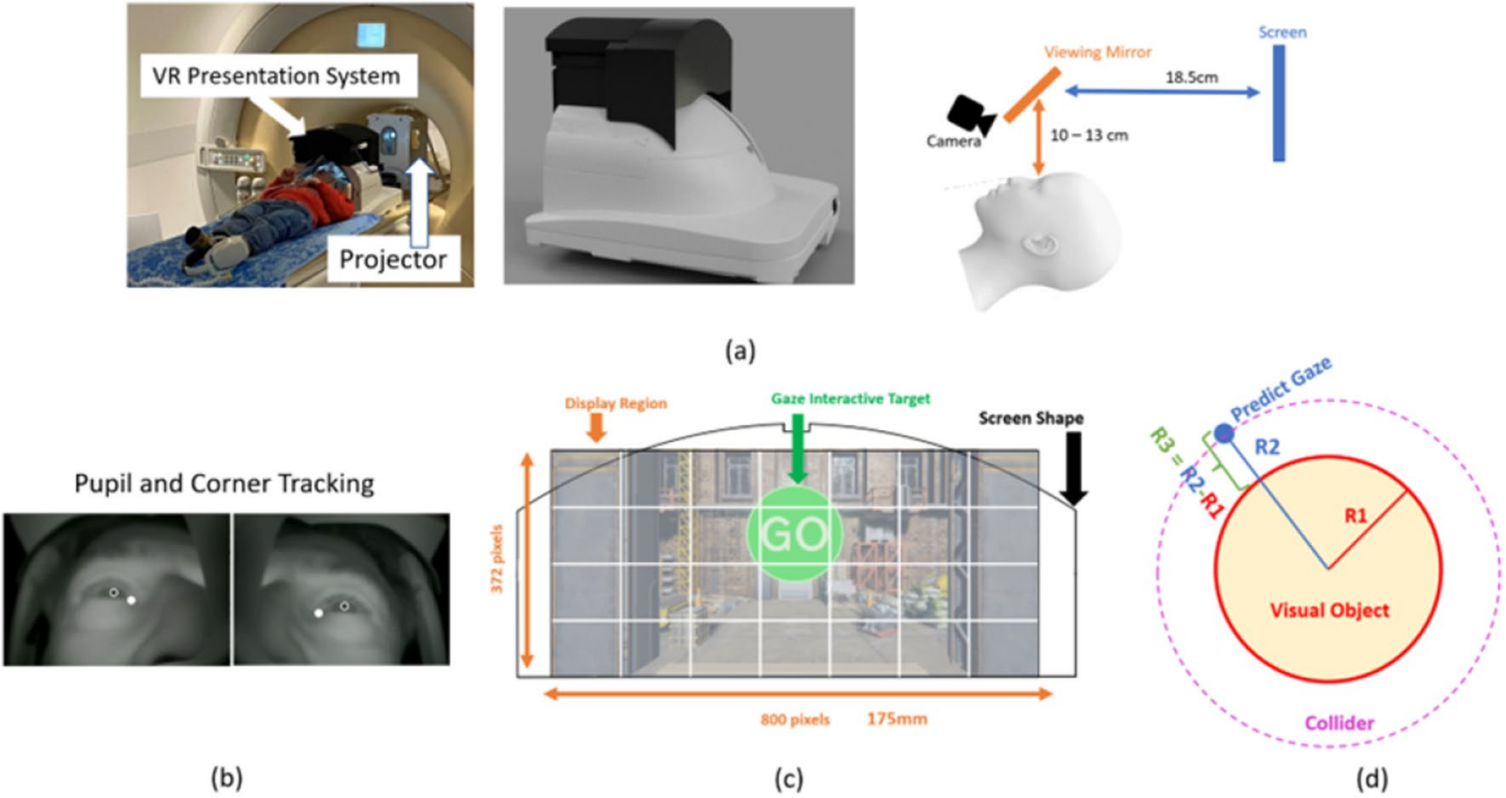
Illustration of the experiment platform, customized VR system design, display region and gaze interactive target modelling. (**a**) From left to right, it presents: a gaze tracking based MRI compatible VR system, the coil mounted VR presentation system design and the visual display system of the presentation system which comprises a screen, a viewing mirror, and infrared cameras for eye tracking. (**b**) The pupil and eye corner tracking result. (**c**) A 800 $\times$ 372 pixels display region is projected onto a curved screen. (**d**) The gaze interactive target consists of a bounding collider and visual object region. The bounding collider is used to capture the gaze overlap. The visual object region is what the subject actually sees on the screen. The gaze prediction error is expressed as percentages of the screen width resolution (in pixels). It is defined as the gaze’s distance relative to the visual object region (donated as R3) divided by screen width (800 in pixel).

The experimental platform used a VR presentation system mounted directly on a standard MRI head coil (Fig. 3a). The VR system includes a display screen (see Fig. 3c) to present projected content and two eye tracking cameras with infrared LED illuminators (12M-I, MRC Systems, Heidelberg) that provide separate video streams for each eye with limited fields of view that include an overlap across the mid-line (Fig. 3b). For the gaze interactive target operation, we adopt a dwell-time based solution. As can be seen in Fig. 3d, when the currently estimated gaze overlaps with a target’s (invisible) bounding collider region, the target’s visual object shrinks (while the bounding collider size remains unchanged). Once the gaze dwell time exceeds a pre-defined threshold (set empirically to 1.8 s), an event associated to this target is triggered (the user achieves their aim), the target disappears and an $({\textbf {e}},{\textbf {t}})$ pair is recorded to be added to the list of values used to create the gaze model. The gaze error is defined as the distance between the predicted gaze location and the visual object boundary (see Fig. 3d). The object boundary rather than its centre is used acknowledging that the gaze can legitimately fall anywhere within visual targets. In our study, the visual targets were designed to occupy a field of view ranging from 2.5 to 4 degrees, with their radius approximately constituting 8% to 12% of the screen width. The viewing distance for each user is determined by the size of the head, so varies across users, but is stable for each user. The viewing distance ranges between approximately 29–33 cm across all subjects.

### User task, data collection and experiment groups

The user task is to utilize gaze to control navigation in the VR system and select content of interest. The design architecture of the VR system follows mainstream system design which consists of explicit calibration, interactive familiarization and a lobby^36^. In the explicit calibration phase, nine point targets are displayed sequentially on the screen using an brief animation to attract and hold attention. Corresponding subject gaze fixation samples are collected, and these are then used to build the gaze model. During the interactive familiarization phase, users are acquainted with fundamental gaze interaction tasks and learn how to navigate within the system. 23 gaze interaction tasks are embedded into a short interactive game which guides the user through a cartoon scene in which they tidy a room by cleaning up rubbish using gaze interactions, before progressing into the lobby (details can be found in the Supplementary Video). The lobby offers a virtual space with visual cues that enable users to navigate to game or cinema sections using gaze control. Two games are offered: a tower defence game, where gaze control is used to halt an onslaught of enemies who arrive at ever faster rates, and a memory card flip game, where gaze control is used to flip cards and match pairs. The movie section features 6 cartoon movies and a channel to access external video resources like YouTube and Netflix, all of which are selected using gaze control.

Data was collected from three groups of subjects using the adaptive gaze estimation framework with or without the initial calibration sequence:

The Explicit Calibration Group comprised seven adults and five children (7 to 13 years). Each subject performed the 9-point explicit calibration, followed by experiencing the interactive VR familiarization, and unrestricted system exploration with the adaptive gaze estimation framework. Time series of $({\textbf {e}},{\textbf {t}})$ data were collected from all phases of operation throughout each session. The global relative coordinate system was used for the interactive operation phase. If the subject was unable to follow the calibration procedure, resulting in pupil recordings taken when they were not looking at the correct target, the calibration procedure was repeated with encouragement and verbal instructions as needed.

The Calibration-Free Group comprised 14 adults. Their engagement began directly with the interactive VR familiarization without explicit calibration. This was subsequently followed by unrestricted system exploration. The corner relative space was used and a prior model was constructed using the data garnered from the Explicit Calibration Group.

The Instant Gaze Group included 8 subjects ( six adults, two 2 years old children). Similar to the Calibration-Free Group, they commenced directly with the interactive familiarization part without explicit calibration, followed by unrestricted system exploration. The prior model was formed from data sourced from the Explicit Calibration Group. However, the distinctive feature of this group is the gaze estimation, which is based on local relative space (instant gaze).

Before introducing the experiment details, we define two key terms: (1) “Full trial samples” refers to the complete time series data acquired for a given subject, including successful explicit calibration if performed, and all data obtained using gaze to control the system. (2) “Calibration pattern samples” refers to paired target and eye feature data collected from the 9 target locations used during explicit calibration. Interactive targets located at these same locations are also embedded in the familiarisation phase so that all subject examinations contain these data.

## Experiments

We conducted five experiments:

### Calibration effectiveness experiment

To find out the effectiveness of the calibration procedure by comparing the user task success rate of the three groups. If a calibration procedure failed (due to various factors^2^), the subject was asked for feedback, given supportive instruction and instructed to repeat the procedure until success, defined as achieving a calibrated gaze model with sufficient accuracy to initiate operation of the adaptive gaze system, was achieved. All subjects used the system without any verbal instructions or tutorials about eye tracking control.

### Prior model performance experiment

To analyse performance as a function of the number of subjects included in the prior model. This experiment is based on the data collected from the Explicit Calibration and Calibration-Free Groups. The following steps were performed: (a) pick full trial samples from n subjects (n $=$ 1,2,3...22) to build the prior model, (b) run the gaze estimation procedure (with and without adaptive model update) on the full trial samples for the remaining $23 - n$ subjects using global, corner and local space based methods respectively, (c) record the gaze error for each fixation for the remaining $23 - n$ subjects, and (d) for each of the remaining $23 - n$ subjects, record the 95th percentile gaze error for the first and second halves of the full trial samples.

### Arbitrary initial target experiment

To determine the robustness of instant gaze control when using an arbitrary target as the initial selection target. This experiment is based on the data collected from the Explicit Calibration and Calibration-Free Groups. The idea is to choose an arbitrary target from the subject’s full trial samples and use that target as the initial selection target. For each subject, the following steps were performed: (1) based on the target display order (see Fig. 5a), we create 22 test sequences of eight targets (see Fig. 5b). The first target in each test sequence is used as the initial selection target. (2) Run gaze estimation algorithm with adaptive model update on the test sequence using the local relative space method and record the gaze error for each target in the test sequence.

#### Instant gaze effectiveness test on retrospective data

To study the performance of instant gaze using existing data. This experiment is based on the data collected from Explicit Calibration and Calibration-Free Groups. Based on the “prior model performance experiment”, a sufficient number of subjects, $n_s$, for acceptable model performance is selected and different ways of building and deploying the prior model explored: (1) using only single subject calibration pattern samples, (2) using the calibration pattern samples of $n_s$ subjects, (3) using the full trial samples of the $n_s$ subjects. With each model, the gaze estimation algorithm was run with and without adaptive update for each subject and the gaze error for every fixation of each subject was recorded.

### Prospective instant gaze effectiveness experiment

To test the performance of instant gaze system using the best performing coordinate model from the outset. Data was collected prospectively from the Instant Gaze Group and analysed for gaze errors when using both static and adaptive gaze models.

## Results

Given the nature of the user tasks-which involve navigation and content selection-the number of gaze samples collected during each trial varied for each subject spanning from 40 to 107 across all participants.

**Figure 4:**
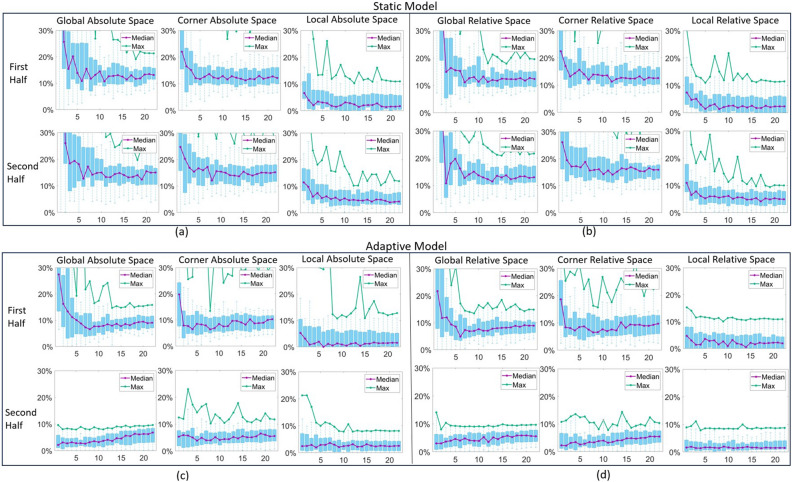
Graphs showing performance as a function of numbers of subjects used to create prior for the different mode of operation with both static models (**a,b**) and with adaptive gaze models (**c,d**). The X-axis represents the number of subjects used to build the prior model. The Y-axis is the gaze error. For each sub-figure, the first row shows the first half of the full trial samples and the second row shows the second half of the full trial samples.

Calibration effectiveness experiment: In the explicit calibration group, we observed that 8 out of 12 subjects failed to achieve successful calibration at least once. All of the children failed at their first attempt, with the subject (aged 7 years) who experienced the most failures having to make four attempts. Explicit calibrations were deemed to have failed if the resulting model produced gaze estimation errors as large or larger than the total screen size. Under these conditions, the subject was unable to initiate the familiarisation phase and could not achieve any useful gaze interactions during free exploration. In the Calibration Free and Instant Gaze groups, all subjects successfully achieved gaze control immediately and finished the user task.

Prior model performance experiment: Fig. 4 illustrates results for the six coordinate system choices: 3 choices of origin each with absolute pupil coordinates given the choice of origin or with pupil position relative to the eye corner feature. In each case the distribution of the 95th percentile gaze error for the first half (upper row) and second half (lower row) of the full trial samples are plotted as the number of subjects used to create the prior model is increased (horizonal axis). All possible choices of the current number of subjects chosen from the available 23 were tested with the errors calculated using all the remaining subjects. In Fig. 4a, the static model’s general performance is worse than the adaptive model displayed in Fig. 4b. In Fig. 4b, from the first half’s local relative space performance, it can be seen that the prior model’s performance stops improving after including full trial samples from 6 to 7 subjects (approximately 220–280 gaze samples). The local relative space representation generally outperforms the global and corner space representations in both first and second halves of the trials. For the second half, the local relative space performance is better than the other rest methods. The overall performance of the second half surpasses that of the first half, owing to the implementation of adaptive model updates. Generally, the analysis demonstrates that the prior model should incorporate full trial samples from at least 6–7 subjects, and that the adaptive/static local relative space representation’s performance surpasses the other representations.

**Figure 5:**
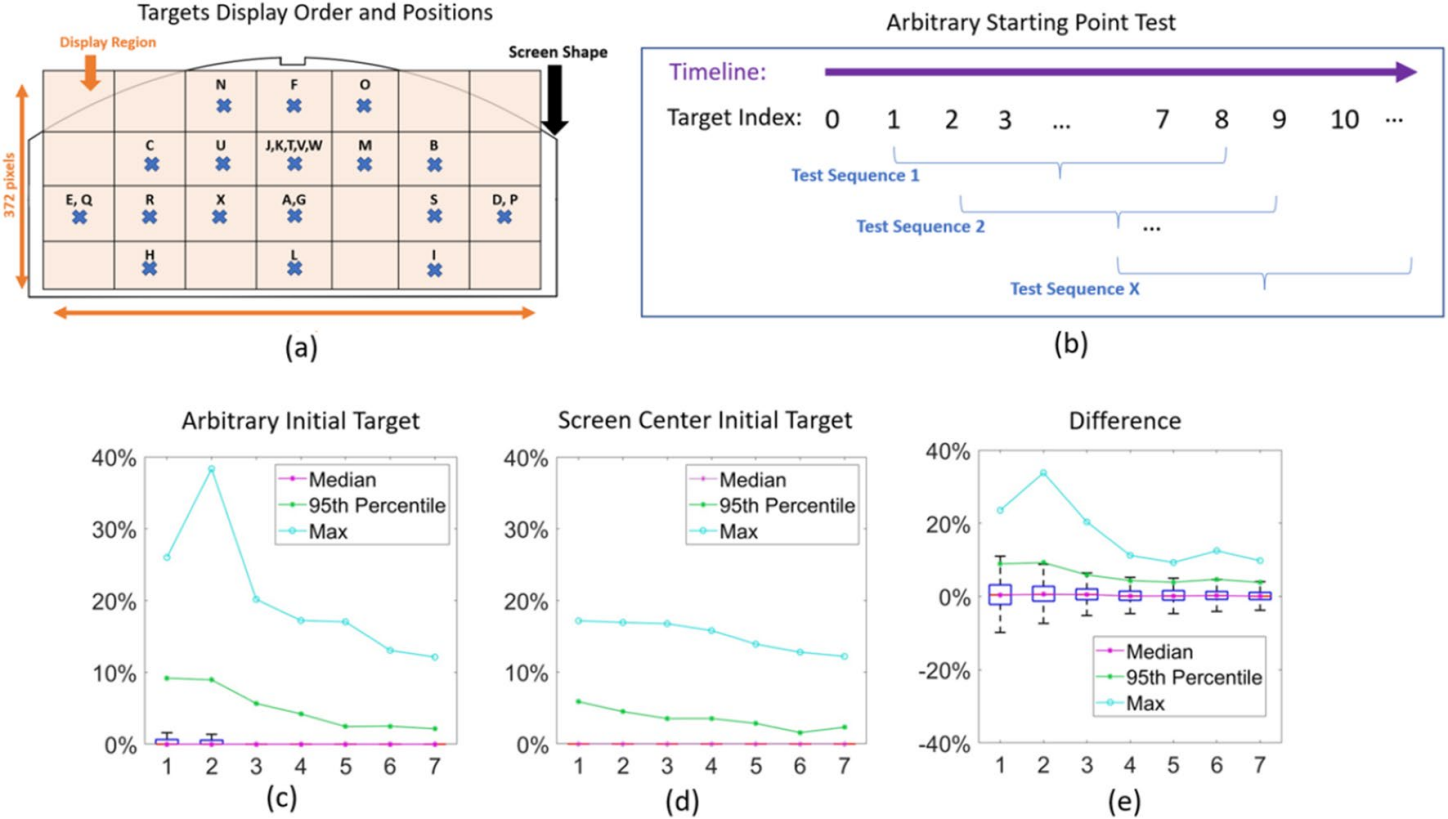
Arbitrary initial selection target experiment. (**a**) The display order (coded by capital letters in alphabetic order) and positions (marked by the blue cross) for the first 23 targets. The screen display area is divided into a 4-row by 7-column grid, with targets appearing at the center of their respective grids. (**b**) Visualization of the test sequences, which have a length of 8, with each new sequence starting one target later. (**c,d**) Test sequence performance graphs for all subjects and sequences (23 subjects $\times$ 22 sequences) showing various error measures as through the sequence targets using an arbitrary starting point in (**c**) and the screen center target in (**d**). (**e**) The difference between (**c**) and (**d**).

Arbitrary initial target experiment: it can be seen from Fig. 5c that the 95th percentile gaze error is smaller than 10% and 75th percentile is much smaller, confirming that the local relative space based method supports instant gaze interaction no matter where the initial selection target is. To compare the results obtained using the screen centre as the initial target, we conducted the same experiment depicted in Fig. 5c while substituting the reconstructed eye features associated with the screen centre with the actual recorded eye features related to the screen centre (Fig. 5d shows the result). In Fig. 5e, we compare the difference between (c) and (d). The error difference distribution shows very little offset (it is centred at zero) which means two methods have similar performance. However, using an arbitrary location for the initial target leads to larger maximum error than when using a target located at the screen centre.

**Figure 6:**
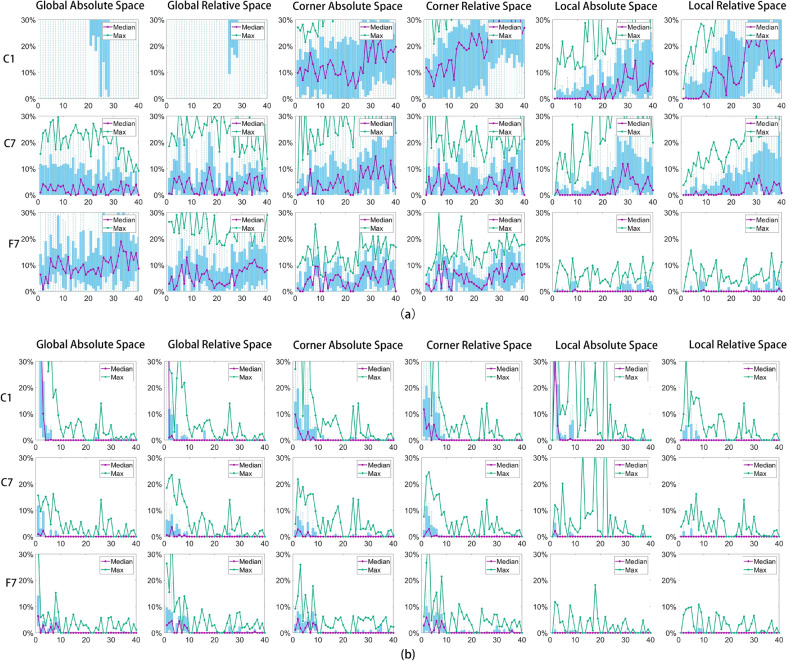
Instant gaze control performance comparison. (**a,b**) The static and adaptive gaze estimation performance respectively, both based on prior models built from: calibration pattern samples from a single subject (first row, labeled as (C1)), calibration pattern samples from seven subjects (second row, labeled as (C7)) and seven subjects full trial samples (third row, labeled as (F7)). The subjects are selected from training groups.

Figure 6a, b report results for instant gaze performance (distributions of gaze errors) for models when using prior models based on the calibration pattern samples from single subject (first row), calibration pattern sample from seven subjects (second row) and full trial samples from seven subjects (third row). The adaptive gaze estimation’s performance (Fig. 6b) is clearly superior to that of static gaze estimation (Fig. 6a). With the increase of gaze sample diversity (or subject number) and amount, the performance of the adaptive and static local space based method improves much more significantly than global space and corner space based methods. Although the performance of the adaptive and static global space and eye corner space based methods also improve with the increase of gaze samples diversity and amount, they do not achieve instant gaze control because of the large initial gaze error. For the adaptive local relative space based method (see the last row and last column of Fig. 6b), the maximum gaze error is less than 10% and the 75th percentile error is less than 4% throughout the whole trial for each subject. Thus local relative space based adaptive gaze estimation enables users to achieve instant gaze interaction. The local absolute space based adaptive gaze estimation max error is worse than the local relative based method. From Fig. 6a, although the max and 75th percentile error of the static local space based methods are worse than those of the adaptive local space based method, they still outperforms the static global and corner space based methods.

**Figure 7:**
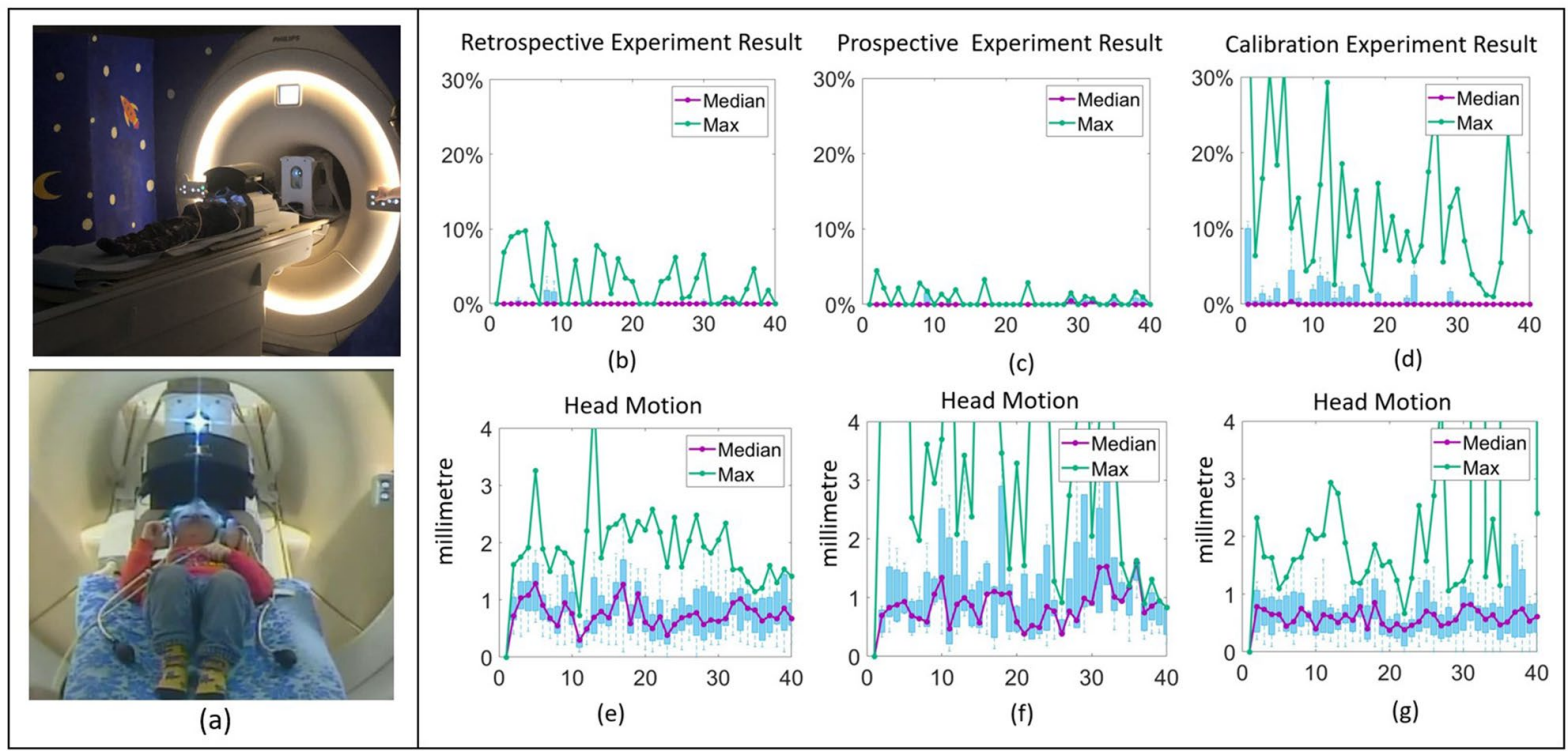
Prospective experiment. (**a**) The youngest participants (two 2-year old children) using the system. (**b**) Performance data showing gaze estimation error determined from retrospective analysis of data collected using the global and corner relative coordinate systems. (**c**) Gaze estimation error during prospective operation of the system using a fully local coordinate system. (**d**) Gaze estimation error during explicit calibration based test in the calibration effectiveness experiment. (**e**) Changes in head position for the trial data in (**b**), and (**f**) changes in head postion for the prospective experiments reported in (**c**). (**g**) Changes in head position for the explicit calibration test in (**d**). Changes in head position are from one fixation sample to the next and are shown in millimetres. In all graphs the horizonal axis is fixation number (a proxy for time during the examination).

Figure 7a shows two 2 year old children using the system. Figure 7b shows the gaze errors for adaptive calibration using the local relative coordinate system on data collected using the global and corner relative coordinate systems and Fig. 7c shows errors for the final optimised system operated prospectively using the local relative coordinate system. Although in both cases, instant gaze is achieved and median errors remain at the 1–2% level (within 0.7^∘^ error), the maximum errors are reduced when the local relative coordinate system is used throughout system operation. By examining Figs. 4 and 6, it is evident that the performance of these methods is worse than the local relative space-based approach. Consequently, the system performance may be adversely affected by the less precise pupil-target correspondences in the ${\textbf {e}}$ data leading to less accurate personalised models. The prospective local based adaptive methodology results in secure operation with sustained low levels of error and good control of maximum error (less than 5% which is equal to gaze error within 1.7^∘^ ). Figure 7e, f illustrate the head position changes throughout system operation, demonstrating the robustness of the adaptive local relative space-based gaze estimation to head motion. The head position is estimated by calculating the average position of the left and right eye corners. Translation of the average eye corner positions therefore here serves as a proxy for all changes in head pose/position. Note that in this data there were larger head movements in the prospective operation experiment (Fig. 7f), yet performance in terms of gaze tracking error was still best (Fig. 7c). Comparison of our method with a traditional calibration based test shows that although the head motion range in the traditional calibration test (see Fig. 7g) turned out to be smaller than in the prospective experiment in Fig. 7f (Mann–Whitney U test result: p-value<10^-15^), the gaze performance in the traditional calibration test (see Fig. 7d) is much worse than in the prospective experiment in Fig. 7c (Mann–Whitney U test result: p-value<10^-4^). This clearly demonstrates the robustness of the proposed method compared to traditional calibration. It is worth noting that the youngest participants (see Fig. 7a) were able to continue performing the user tasks for at least 20 min with the MRI scanner running.

## Discussion

In this study we have explored how to achieve stable and accurate gaze estimation without the need for an explicit calibration sequence to initialise the system. The result is a gaze interaction HCI that starts to operate as soon as the feature detection software detects the eye and eye corner features that are to be tracked. From the subject’s perspective, they see an opening screen with a single target that responds to them looking at it. This visual feedback informs them that the system is operating. The recorded initial eye and eye-corner features, which are captured when the subject looks at the initial target, are sufficient to customise the model. This immediately results in a capability that can operate with a static model and can be used to passively track the subjects gaze in real time. More precise performance that is also more robust to head motion is achieved by continuing to collect eye feature data paired with the current target location as the subject continues to use the gaze interaction system. This allows the gaze model to be progressively refined and enables stable gaze control to be maintained even when, as is the norm, the subject’s head position varies. In our testing the subjects did not need any instruction to use the system despite never having experienced it before. The user task included free exploration of games and a choice of videos to watch. Session length was determined by the subject running out of materials to engage with. In no case did the subject request to terminate the session because the system failed or because they experienced strain in operating the gaze control.

The calibration effectiveness experiment demonstrated that our method outperforms the traditional calibration procedure. Most subjects in the explicit calibration group failed to achieve successful calibration at least once, while all subjects in the calibration-free and instant gaze groups were able to achieve gaze control immediately and complete the user task. This suggests that the proposed method is both reliable and usable at least for the specific VR system used in testing.

The prior model performance experiment showed that the local relative space representation generally outperforms the global and corner space representations, both in terms of gaze error and number of subjects needed to build an effective prior model. This supports our hypothesis that representing eye features in a local relative coordinate system can reduce data variation between subjects and that this improves the performance of gaze estimation. As shown in Fig. 4d, the model’s performance ceases to improve when incorporating additional data beyond 6–7 subjects. This threshold is likely a result of the low dimensionality of the quadratic polynomial regression gaze model. Our findings is in keeping with work which has investigated the effect of order and forms of the polynomial regression function in gaze estimation, with the best accuracy that can be achieved found to be 0.5^∘^^2,37^ provided that the subject’s head remains as still as possible. In comparison, our system demonstrated an error of 0.7 degrees at the 75 percentile in the small head motion regime tested. An analysis^14^ evaluating more than 400,000 mapping functions concluded that employing polynomials of higher orders does not yield significant enhancements in performance.

The instant gaze effectiveness test on retrospective data demonstrated the superior performance of instant gaze using existing data. The experiment revealed that with the increase of gaze sample diversity and quantity, the performance of the local relative space based method improved significantly. The prior model performance experiment shows that only a small number of subjects are needed to build effective prior models; in the case of our described system, 7 subjects were sufficient.

The arbitrary initial target experiment confirmed that the gaze error remained small for all tested locations of the initial selection target for instant on operation. This demonstrates the flexibility and adaptability of our method.

The prospective instant gaze effectiveness experiment showed that the proposed method can effectively reduce gaze error and maintain a consistent level of accuracy throughout prolonged interaction sessions. Through comparison with use of traditional calibration, it was observed that head motion in the traditional calibration test was smaller when compared to the prospective experiment. A possible contributing factor for this is that the subjects who failed the calibration procedure (8 out of 12 failed) were subsequently instructed to keep their head still for a repeat calibration. The early phase error of the traditional calibration test (Fig. 7d) is large which is aligned with existing evidence^2^ which suggests that traditional calibration procedures are sensitive to head movements. The challenge that some subjects clearly experienced in successfully completing the conventional calibration supports our motivation for developing the system. This comparative analysis firmly positions the proposed method as a more user-friendly alternative, offering a seamless experience from the beginning of the interaction and affirming the effectiveness of the instant gaze method.

A limitation of the current work stems from the use of the eye corner as a proxy to quantify head motion. This was highly effective for the VR system under test, which is designed for use during MRI examination using a head coil with subjects in a supine position. In this application (and therefore in our studies), the magnitude of head motion is constrained with a maximum translation of 30mm.

Clearly in other settings much larger head motions might be feasible or even common and this would likely require use of more comprehensive feature sets to characterise changes in head pose. Exploration of regimes with larger head motions is beyond the scope of this paper, but is clearly a highly desirable generalisation for key populations such as children or those with cognitive or physical disabilities.

A significant aspect of our instant gaze approach is that it can provide the user with an instant gaze interaction experience with just a single target interaction. Although the initial gaze estimation may not be highly precise (see Figs. 6 and 7), the local relative space based eye feature representation still provides the user with an immediate interaction capability. From the GUI design perspective, our method is better suited to be used for navigation task as a starting point (which is how it is used to initiate system navigation in our MR compatible VR system, see Supplementary Video). To adopt our instant gaze control framework, a GUI with an adaptive design based on the gaze error is required. An initial target is also needed from the start of system use. The beginning target layout resolution should be sparse. With the improvement of gaze performance, more dense, higher resolution target layouts can be used. For example, the max gaze error curve shown in Fig. 7b supports 2 (row) $\times$ 4 (column) target layouts on our screen which has a resolution of 800 $\times$ 372 pixel. Although these layouts remain quite sparse, they are sufficient for many navigation based tasks. For example, most VR/AR systems have an introduction or tutorial stage, in which the user needs to focus on a limited number of targets (1–2 targets) to familiarise themselves with the system. Those targets are ideal for gaze interaction and adaptive update. Comparisons with existing gaze estaimation/tracking methods were introduced in detail in the related works section.

In the discussion of gaze control systems, addressing the concerns of false positives (unintentional successful target gaze registration) and positive errors (intentional gaze by a participant that fails to register) is critical. These are common challenges that can damage efficacy and particularly impact on the user experience of such systems. Our VR system exclusively employs navigation-focused tasks that inherently demand intentional selection by the user. Specifically, the system will not progress in the absence of a gaze initiated event, thereby minimizing unintended interactions. To minimise unintended user interactions, we use a dwell time-based mechanism and provide feedback to the user that they are initiating an event. This enables them to shift their gaze if they do not wish to make the current selection. This approach guarantees that only conscious gaze interactions are registered, significantly reducing the likelihood of false positives. From the GUI design perspective, we recognize the potential for initial low accuracy due to the adaptive nature of our gaze estimation method. To address this, we have adopted a progressive design strategy, starting with few and well separated targets, as discussed above. This design choice is aimed at mitigating false positives, especially in the initial phases of use. As the system adapts and accuracy improves, the interactive areas are gradually scaled down, taking advantage of more precise gaze control to reinforce the user’s perception control with the ability to intentionally initiate or not to initiate interactions by making the trigger features have precise visual confirmation.

Our empirical observations support the effectiveness of these strategies. Throughout the normal use of the system, there were no instances observed where participants intentionally diverted their gaze away from the intended targets. All participants successfully navigated and selected content of interest, demonstrating the system’s ability to accurately capture and respond to intentional gaze interactions. Although the potential for errors in target selection exists, either an accidentally trigger or a failure to achieve a trigger when the subject thinks they are gazing at a target, such occurrences were not encountered among our subjects. Errors hinder system navigation and cause user frustration, so it is a central requirement that they are avoided. The test results in our testing phase affirm the system’s capability to precisely capture intentional gaze interactions and its resilience against common gaze control system issues.

In general, from the perspective of achieving instant and user-friendly gaze control experience, the proposed method is unsurpassed by existing implicit calibration methods in procedure length and usage complexity. There is no need for extra hardware or for protracted support/set-up tasks. From the perspective of the gaze model, it provides a simple way to construct eye features which is less influenced by the data variation than the global based methods. Although we only demonstrated the method using polynomial regression based gaze estimation, the proposed approach has clear potential to be adapted for other gaze estimation methods which rely on diverse eye features as input.

## Conclusion and future works

In this paper, we proposed a novel local relative space based gaze estimation method which can provide instant gaze information for human computer interaction under small head motion. Our tests showed that use of a relative space eye feature construction method which models gaze directional changes and head motion within distinct local relative spaces, is robust to pose variability and individual subject differences. By working with an adaptive gaze model framework, the method can provide instant and continuously reliable gaze information. We proved the effectiveness of the method through a gaze-controlled Virtual Reality based patient entertainment system for Magnetic Resonance Imaging (MRI) exams. The results shows a maximum error within 1.7^∘^ and 75th percentile error within 0.7^∘^, and that these could be maintained for extended examinations, remaining reliable and accurate even when subjects moved their heads. The instant on feature proved to be highly appealing to our test subjects who were able to operate the system immediately after being placed into a position. In contrast, the volunteers who had to perform explicit calibration procedures as part of our testing schedule clearly found that part of the experience both boring and frustrating.

The method explored here is designed for small head motion and the eye tracking is based on images collected under infrared illumination. In the future, we will extend the current method for large head motion and general webcam based use cases. This would make the method more accessible and versatile, catering to a wider audience and more diversity of devices.

We hereby affirm that all subjects participating in our experiment have given their informed consent for the publication of their identifying information and/or images in an online open-access publication.

### Supplementary Information

Supplementary Video.
